# Model-based understanding of single-cell CRISPR screening

**DOI:** 10.1038/s41467-019-10216-x

**Published:** 2019-05-20

**Authors:** Bin Duan, Chi Zhou, Chengyu Zhu, Yifei Yu, Gaoyang Li, Shihua Zhang, Chao Zhang, Xiangyun Ye, Hanhui Ma, Shen Qu, Zhiyuan Zhang, Ping Wang, Shuyang Sun, Qi Liu

**Affiliations:** 1 Department of Endocrinology and Metabolism, Shanghai Tenth People’s Hospital, Bioinformatics Department, College of Life Science, Tongji University, Shanghai, China; 2 Department of Ophthalmology, Ninghai First Hospital, Ninghai Zhejiang, China; 3 Tongji University Cancer Center, Shanghai Tenth People’s Hospital of Tongji University, Shanghai, China; 4 School of Medicine Tongji University, Shanghai, China; 5 Institute of Applied Mathematics, Academy of Mathematics and Systems Science, Beijing, China; 6 Shanghai Chest Hospital Shanghai Jiaotong University, Shanghai, China; 7 School of Life Science and Technology ShanghaiTech University, Shanghai, China; 8 Department of Oral and Maxillofacial-Head Neck Oncology, Shanghai Ninth People’s Hospital, College of Stomatology, Shanghai Jiao Tong University School of Medicine, Shanghai, China

**Keywords:** Bioinformatics, Computational biology and bioinformatics

## Abstract

The recently developed single-cell CRISPR screening techniques, independently termed Perturb-Seq, CRISP-seq, or CROP-seq, combine pooled CRISPR screening with single-cell RNA-seq to investigate functional CRISPR screening in a single-cell granularity. Here, we present MUSIC, an integrated pipeline for model-based understanding of single-cell CRISPR screening data. Comprehensive tests applied to all the publicly available data revealed that MUSIC accurately quantifies and prioritizes the individual gene perturbation effect on cell phenotypes with tolerance for the substantial noise that exists in such data analysis. MUSIC facilitates the single-cell CRISPR screening from three perspectives, i.e., prioritizing the gene perturbation effect as an overall perturbation effect, in a functional topic-specific way, and quantifying the relationships between different perturbations. In summary, MUSIC provides an effective and applicable solution to elucidate perturbation function and biologic circuits by a model-based quantitative analysis of single-cell-based CRISPR screening data.

## Abstract (web summary)

Single-cell CRISPR screening combines pooled CRISPR screening with scRNA-seq analysis to expand the resolution power of genetic screening. Here, the authors develop MUSIC, a computational pipeline for analyzing single-cell CRISPR screening data.

## Introduction

Pooled CRISPR knockout screening is a powerful technique for evaluating the biologic function of genes. This technique, however, only recognizes genes with very distinct phenotypes, such as those that affect cellular growth substantially or can be detected with antibodies or fluorescent protein reporters directly, which limited its ability to detect other genes with subtle phenotypes^1–3^. Recently described novel methods, i.e., single-cell-based CRISPR knockout or knockdown screening (independently termed Perturb-Seq^4,5^, CRISP-seq^6^, and CROP-seq^7,8^), combine pooled CRISPR screening with single-cell RNA-seq to investigate functional CRISPR screening in a single-cell level. These screening methods make it possible to implement large-scale gene perturbation study in a more elaborated way.

The key technical innovation for single-cell CRISPR screening including Perturb-Seq^4,5^, CRISP-seq^6^, or CROP-seq^7,8^ lies in modifying the lentiviral vector to allow for identification of the sgRNA in a single cell from deep-sequencing of mRNAs (polyadenylated RNA fraction)^3^. By taking advantage the innovation in performing mRNA-seq on individual cells, large-scale cells with distinct perturbations within a heterogeneous cell population can be investigated^3,9^.

Several computational challenges exist in the analysis of such single-cell CRISPR screening data: (1) Data sparsity and noise. Single-cell RNA-seq data is sparse^10,11^. In addition, both single-cell RNA-seq data and pooled CRISPR screening data are inherently noisy^12,13^, and this is further exacerbated by their combination. Efficient data filtering and normalizing are needed to meet these challenges. (2) The sgRNA perturbation and off-target effect should be carefully investigated when linking such perturbations with the gene expression readout^14,15^, particularly for heterogeneous cell-to-cell comparisons. (3) Quantitative and parallel estimating and prioritizing the effect of each perturbation and their relationships on different cells with cellular heterogeneity and technical complexity is required, and (4) Intuitively visualizing the perturbation results at a large-scale heterogeneity cellular level is needed. To this end, we developed MUSIC, which is an integrated tool for model-based understanding of single-cell CRISPR screening. This is an easy-to-use and model-based integrated analytical tool designed specifically for single-cell CRISPR screening data analysis.

## Results

### General pipeline of MUSIC

**Fig. 1:**
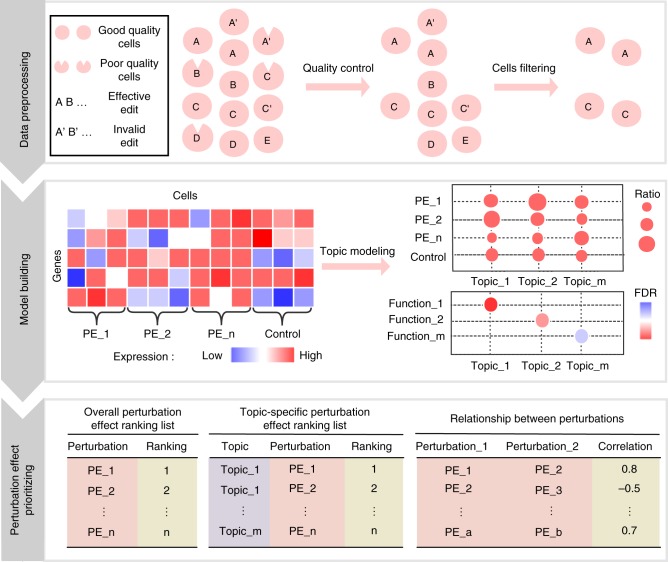
General workflow of MUSIC. MUSIC comprises three steps for single-cell CRISPR screening data analysis: data preprocessing, model building, perturbation effect prioritizing. In the 1st step, besides the conventional considering of cell quality, several specific factors existed for single-cell CRISPR screening are also considered. These factors are the ratio of nonzero perturbed expression value in all cells, sgRNA efficiency and the minimal perturbed cell number per perturbation. In the 2nd step, MUSIC applies a topic model-based computational framework to derive the functional topics of each cell (including controls) with specific perturbation (PE, perturbation). In the 3rd step, MUSIC quantitatively estimates and prioritizes the individual gene perturbation effect on cell phenotypes from three different perspectives, i.e., prioritizing the gene perturbation effect as an overall perturbation effect, or in a functional topic-specific way, and quantifying the relationships between different perturbations

MUSIC comprises three steps for single-cell CRISPR screening data analysis (Fig. 1): data preprocessing, model building, and perturbation effect prioritizing.

In the first step (Fig. 1 and see Methods), besides the routine quality control and data normalization processes applied in single-cell RNA-seq analysis, MUSIC also applied a data imputation step (achieved by SAVER^16^) to improve the data quality. In addition, MUSIC addresses two issues that should be taken into account for such a novel data type: (1) Filtering perturbed cells with invalid edits; (2) Filtering perturbations according to a minimal number of cells per perturbation.

**Fig. 2:**
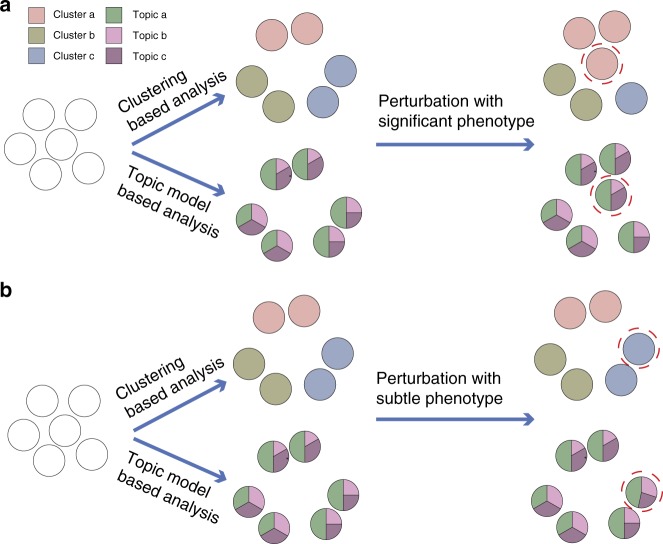
Comparisons between traditional clustering based analysis and topic model  based analysis for single-cell CRISPR screening data. **a** Difference between traditional clustering based analysis and topic model-based analysis for single-cell CRISPR screening data when a perturbation has a significant phenotype on the cells. Both analyses can detect such phenotype change (see the cell sample with red dotted line). **b** Difference between traditional clustering-based analysis and topic model-based analysis for single-cell CRISPR screening data when a perturbation has a subtle phenotype on the cells. Topic modeling calculates a topic probability profile for each sample while traditional clustering just makes a hard assignment of the sample to each cluster. Therefore, in this way, topic-model-based analysis can detect such phenotype change based on the change of topic probability profile with and without perturbation, while traditional clustering based analysis failed to detect such subtle phenotype change (see the cell sample with red dotted line)

Second, MUSIC builds a computational framework based on Topic Models to handle single-cell CRISPR screening data (Fig. 1 and see Methods). The concept of topic models was initially presented in the machine-learning community^17^ for discovery of hidden semantic structures in a text body and has been successfully applied to gene expression data analysis^18–20^. Intuitively, given that a document is about a particular topic, one would expect particular words to appear in the document more or less frequently. The topics generated by topic modeling are represented by class of words with similar sematic meanings. A topic model is a probabilistic framework formulated on the investigation of the giving documents and discovering their topic profiles based on such word frequency representations. By analogy to the single-cell CRISPR screening data, a single cell with perturbation can be taken as a document. The gene expression is analogous to the word frequency in the document. A topic here represents a specific biological function associated with a group of highly differential expressed genes. Therefore, a topic model applied here allows us to examine a set of cells with perturbations and discover, based on the gene expression in each, what the perturbation induced biological functions might be. Two key advantages of the topic model applied here are: (1) it allows each perturbed sample to process a proportion of the membership in each functional topic rather than to categorize the sample into a discrete cluster. Such topic profile, which is derived from large-scale cell-to-cell different perturbed samples, making the following ranking of perturbation impact straightforward and quantitative. As can be clearly illustrated in Fig. 2, compared with traditional clustering, which makes a hard assignment of cells into different subclasses, topic modeling just calculates a topic probability profile for each sample rather than assigning it into subclasses. (2) Topic modeling is sensitive to detect subtle phenotype changes based on the change of topic probability profile with and without perturbation, while traditional clustering generally failed to detect such subtle phenotype changes, which widely exist in single-cell CRISPR screening data (Fig. 2).

In addition, MUSIC addresses several specific issues when applying the topic model to this specific data type: (1) The distribution of topics between cases and controls is affected by the ratio of their sample numbers, and such a sample imbalance issue is addressed by the bootstrapping strategy when prioritizing the perturbation effect (see Methods). (2) The optimal topic number is automatically selected by MUSIC in a data-driven manner (see Methods).

Finally, with the topic-model-based perturbation analysis, MUSIC can quantitatively estimate and prioritize the individual gene perturbation effect on cell phenotypes from three different perspectives (Fig. 1 and see Methods), i.e., prioritizing the gene perturbation effect as an overall perturbation effect, or in a functional topic-specific way, and quantifying the relationships between different perturbations.

### Evaluating the performance of MUSIC

**Fig. 3:**
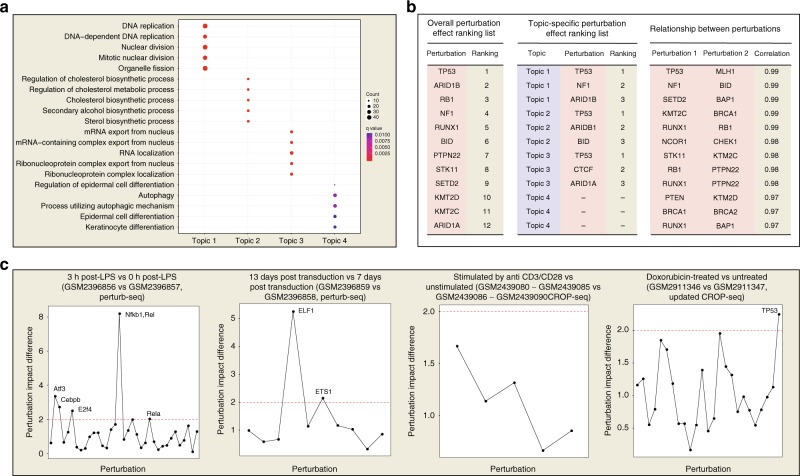
An illustration result of MUSIC for single-cell CRISPR screening data analysis. We take the dataset of MCF10A cells treated with doxorubicin (GSM2911346) by the updated version of CROP-seq^8^ as an example, as illustrated in (**a**, **b**). The overall perturbation effect ranking lists identified by MUSIC were also compared between cells with different treatment, as illustrated in (**c**). **a** The functional annotations of each topic derived from topic modeling for dataset GSM2911346. **b** The overall perturbation effect ranking list and the topic-specific perturbation effect ranking list for dataset GSM2911346. **c** The differences of perturbation impact between different experimental conditions are demonstrated respectively for Perturb-Seq^5^ and CROP-seq^7,8^ data

To evaluate the performance of MUSIC, we made the following two aspects of analysis. We started our study by applying MUSIC to all publicly available 14 sets of single-cell CRISPR screening data, including Perturb-Seq^4,5^, CRISP-seq^6^, and CROP-seq^7,8^ to obtain the analysis results (Supplementary Table 1). For illustration purposes, we took the doxorubicin-treated MCF10A cells^8^ with 29 tumor suppressors perturbed as an example plot (Fig. 3a, b). Detailed analysis results of all the other datasets can be accessed in the supplementary materials (Supplementary Data 1–14 and Supplementary Fig. 1–14).

Then, we compared MUSIC with two other mentioned tools MIMOSCA^5^ and LRICA^4^ (Tables 1 and 2). MIMOSCA is a computational framework to handle multiple input multiple output single-cell data analysis. LRICA is proposed to decipher the driver signal/component of the data by low-rank matrix factorization. Although MIMOSCA and LRICA models were presented in the literatures, they were only developed as the prototypes without executable and user-friendly implementations. In addition, the output of MUSIC is different from these tools and they are not straightforward to be compared. Therefore, we provided the preliminary comparison results in Tables 1–3 for several datasets to indicate the effectiveness of MUSIC.

**Table 1:** Comparisons of detail analysis results between MUSIC and MIMOSCA

Datasets	Technology	Demonstrated perturbation	Output	MIMOSCA	MUSIC
Mouse BMDC (3 h post-LPS, GSM2396856)	Perturb-Seq^5^	*Cebpb*	Overall perturbation effect	—	Rank 2nd
			Topic-specific functional perturbation effect	Immune cells activation	• Immune cells activation^21^ • Cell migration^22^
			Perturbations relationship	• *Cebpb* and *Nfkb1*, *Runx1*, *Irf4*, *Spi1* have opposing effects.	cor(*Cebpb*, *Nfkb1*) = -0.99 cor(*Cebpb*, *Runx1*) = −0.99 cor(*Cebpb*, *Irf4*) = − 0.99 cor(*Cebpb*, *Spi1*) = − 0.96
				• *Cebpb* and *Rela, HIF1a*, *Stat3*, *Junb* have reinforcing activation.	cor(*Cebpb*, *Rela*) = 0.99 cor(*Cebpb*, *HIF1a*) = 0.98 cor(*Cebpb*, *Stat3*) = 0.99 cor(*Cebpb*, *Junb*) = 0.93
Human K562 (7 days post transduction, GSM2396858)	Perturb-Seq^5^	*GABPA*	Overall perturbation effect	—	Rank 2nd
			Topic-specific functional perturbation effect	Mitochondrial function	• Heme metabolic process • Neutrophil activation^35^
			Perturbation relationship	—	cor(*GABPA*, *ELK1*) = 0.89^36^
Human K562 (cell cycle regulators, GSM2396861)	Perturb-Seq^5^	*AURKA*	Overall perturbation effect	—	Rank 1st
			Topic-specific functional perturbation effect	Proliferation	Proliferation
			Perturbation relationship	*AURKA*, *TOR1AIP1*, and *RACGAP1* perturbed similar.	cor(*AURKA*, *TOR1AIP1*) = 0.70 cor(*AURKA*, *RACGAP1*) = 0.85 cor(*TOR1AIP1*, *RACGAP1*) = 0.75

cor(*a*,*b*) represents the Pearson correlation coefficient of topic distribution profile between perturbation *a* and perturbation *b*

**Table 2:** Comparison of detail analysis results between MUSIC and LRICA

Datasets	Technology	Demonstrated perturbation	Output	LRICA	MUSIC
Human K562 (3 UPR related genes, GSM2406677)	Perturb-seq^4^	ATF6, PERK, IRE1α	Overall perturbation effect	—	The three perturbations’ overall perturbation effect ranks 1st
			Topic-specific functional perturbation effect	UPR	• UPR • Apoptosis^23^
			Perturbation relationship	The perturbation of PERK has a greater impact than those of ATF6 and IRE1α.	TPDS(PERK) = 94.0 TPDS(IRE1α) = 23.2 TPDS(ATF6) = 11.0
Human K562 (83 UPR related genes, GSM2406681)	Perturb-seq^4^	*EIF2S1*	Overall perturbation effect	—	Rank 1st
			Topic-specific functional perturbation effect	UPR	UPR
			Perturbation relationship	—	cor(*EIF2S1*, *DHDDS*) = 0.99

cor(*a*,*b*) represents the Pearson correlation coefficient of topic distribution between perturbation *a* and perturbation *b*

TPDS(*a*) represents the impact score to evaluate the overall perturbation effect of perturbation *a*

**Table 3:** Other representative analysis results of MUSIC

Datasets	Technology	Demonstrated perturbation	Output	Original study	MUSIC
Mouse myeloid cell (GSE90486)	CRISP-seq^6^	*Cebpb*	Overall perturbation effect	—	Rank 1st
			Topic-specific functional perturbation effect	Immune cell differentiation	• Immune cell differentiation^24^ • Cell migration^22^
			Perturbation relationship	—	cor(*Cebpb*,*Rela*) = 0.99^25^
Human MCF10A cell (treated with doxorubicin, GSM2911346)	Updated version of CROP-seq^8^	*TP53*	Overall perturbation effect	—	Rank 1st
			Topic-specific functional perturbation effect	DNA replication	DNA replication^37^
			Perturbation relationship	—	cor(*TP53*, *MLH1*) = 0.99
Human Jurkat cell (stimulated by anti-CD3/CD28, GSM2439086~GSM2439090)	CROP-seq^7^	*LCK*	Overall perturbation effect	—	Rank 6th
			Topic-specific functional perturbation effect	TCR signature	leukocyte differentiation
			Perturbations Relationship	*LCK*, *ZAP70*, *LAT* have similar effect on TCR activation signature.	cor(*LCK*, *ZAP70*) = 0.93 cor(*LCK*, *LAT*) = 0.50 cor(*ZAP70*, *LAT*) = 0.78

cor(*a*,*b*) represents the Pearson correlation coefficient of topic distribution between perturbation *a* and perturbation *b*

First, the comparisons between the analysis results of MUSIC and MIMOSCA were presented in Table 1. MUSIC recapitulated the similar findings as those of MIMOSCA, like the perturbation impact of *Cebpb* on immune cell activation^21^. A novel knockout effect on cell migration^22^ was also identified by MUSIC which are consistent with previous knowledge. MUSIC further identified the gene–gene perturbation relationships, like the recognized associations between *Cebpb* knockout and other gene perturbations by the quantitative correlation calculations (Table 1).

Second, similar comparisons between MUSIC and LRICA were presented in Table 2. Again, MUSIC recapitulated similar findings like LRICA. For example, ATF, PERK, and IRE1α are all important proteins related to unfolded protein response (UPR). Original study has indicated that the perturbation of PERK has a greater impact than those of ATF6 and IRE1α. MUSIC recapitulated this finding in a quantitatively way. In addition, a novel perturbation effect for apoptosis function by knockout the three genes simutaneously^23^ was identified, which indicates that in the absence of the three branches of the UPR, K562 cell enhance the positive regulation of apoptosis signal pathway significantly (Supplementary Data 8 and Supplementary Fig. 8).

Finally, analysis of remain datasets also recapitulated original findings or identified novel results. Representative analysis results by MUSIC on remain datasets are shown in Table 3. *MUSIC* recapitulated the similar results as the original findings, such as the perturbations of *Cebpb* has an important influence on immune cell differentiation^24^. MUSIC further identified several novel findings, such as the high correlation between *Cebpb* and *Rela*^25^ perturbations (Supplementary Data 10). MUSIC identified the special response of *TP53* knockout when cells treated with doxorubicin, which is consistent with previous knowledge^26–28^ (Fig. 3c).

### Evaluating the impact of the data preprocessing strategies adopted in MUSIC

Due to substantially noise existed in single-cell CRISPR screening data, MUSIC adopted several data preprocessing strategies (see Methods), which can effectively improve its performance. In this part, we further explored their impact on the outputs of MUSIC from the following three aspects.

**Fig. 4:**
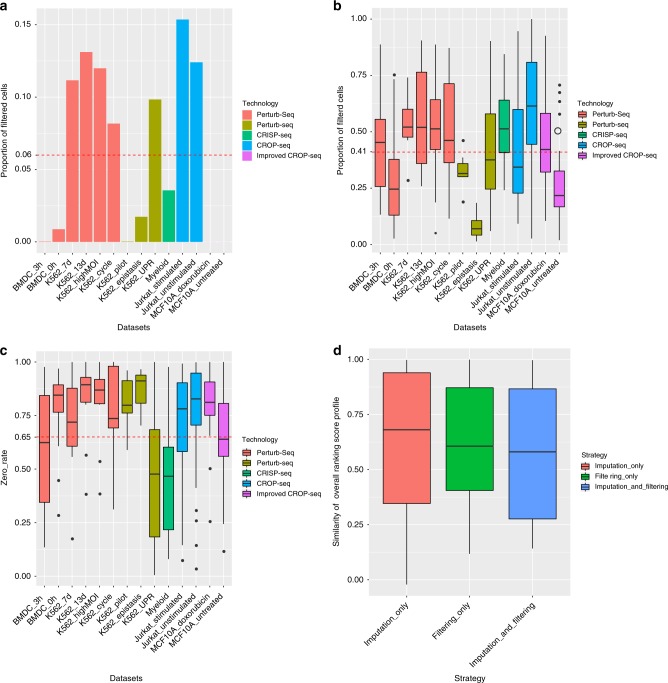
Evaluating the impact of the data preprocessing strategies adopted in MUSIC. **a** The proportion of filtered cells by quality control for all datasets. The red dash line represents the mean of the data. **b** The proportion of filtered cells by filtering low efficiency sgRNA for all datasets. The red dash line represents the mean of the data. **c** zero_rate plot of all knockouts/knockdowns in all datasets. The red dash line represents the mean value of all the knockouts/knockdowns zero_rates. **d** Comparisons of overall perturbation effect ranking with or without imputation/filtering for all the available datasets

First, we provided an overview information on how many cells are filtered from the datasets in the data preprocessing. A statistic summary of the proportion of filtered cells by quality control is shown in Fig. 4a, indicating that an average of 6% of cells are filtered. A statistic summary of the proportion of filtered cells by filtering low efficiency sgRNA is shown in Fig. 4b (Supplementary Data 15). It can be seen that this step filtered an average of 41% cells and these ratios are different in different datasets and  techniques. It should be noted that prior study already indicated the single-cell CRISPR screening technique is very noisy, 20–30% of the cells with a detected sgRNA show a wild-type phenotype^29,30^ and these cells should be filtered.

Second, since the single-cell CRISPR screening data are noisy and zero-inflated, we provided a statistic to show how frequently genes have a zero expression value across all cells. And we demonstrated that our filter strategy will not remove lowly expressed while functional genes like transcription factors. To this end, for all 326 knockouts/knockdowns in all 14 datasets, we calculated their proportion of zero expression values in all cells, which is denoted as the zero_rate of these genes (Fig. 4c and Supplementary Data 16). It is found that that our filtering strategy successfully filters *CDKN2A* in doxorubin-treated and untreated MCF10A cell^8^, which is expected since MCF10A breast epithelium cells carry a deletion of the *CDKN2A* locus. Then only two other genes were filtered. These genes are *PTPRD* in doxorubin-treated MCF10A cell^8^ and *IER3IP1* in K562 cell^4^, probably due to the noise existed in these datasets. These genes are not transcription factors, and all the functional transcription factors are kept to be unaffected. To further evaluate the impact of this filtering on the results of MUSIC, we also performed a test to check what occurs if MUSIC removed this filtering step. We rerun MUSIC and compared the overall perturbation effect ranking with or without zero expression filter for the corresponding three affected datasets (doxorubin-treated and untreated MCF10A and K563 cell). More specifically, we normalized the overall ranking score (see the section of Obtaining the overall perturbation effect ranking list in Methods) in the obtained ranking list calculated with or without zero expression filter. Then we calculated the Pearson correlation coefficients of the normalized overall ranking score profiles with or without zero expression filter. The Pearson correlation coefficients calculated above were 0.99 for doxorubin-treated MCF10A, 0.93 for untreated MCF10A, 0.98 for K562 cell, respectively. Taking together, these results showed that the filtering of zero expression will not induce substantial changes on the overall rankings, which means that the filtering of the corresponding knockouts generally keeps other knockouts or knockdowns unaffected.

Third, we evaluated the impact of imputation and filtering strategies in the data preprocessing step on the final perturbation ranking results. To this end, we took a group of genes tested by Perturb-Seq^5^ as a benchmark, which indicated that *Cebpb* has the strong reinforcing effect on *Rela*, *Hif1a*, *Stat3* and *Junb*, while keeps the strong opposing effect on *Nfkb1*, *Runx1*, *Irf4* and *Spi1*. The relationships available for these genes are so evident that it is ideal to be taken as a golden standard. As shown in Supplementary Table 2, a comparison with or without imputation/filtering were performed on this dataset. It can be seen clearly that imputation and filtering as a whole can uncover such strong positive and negative correlations correctly and accurately. We further made a global evaluation to access the overall impact of the data preprocessing on all the datasets (Fig. 4d). In this study, the overall impact is calculated as the overall perturbation effect ranking correlation with or without imputation/filtering for all the 14 datasets (Supplementary Data 17). More specifically, we first normalized the overall ranking score (see the section of Obtaining the overall perturbation effect ranking list in Methods) in the obtained ranking list calculated with or without imputation/filtering. Then we calculated the Pearson correlation coefficients of the normalized overall ranking score profiles with or without imputation/filtering. The bar plots of such similarity comparisons are shown in Fig. 4d, indicating that how the imputation_only, the filtering_only or their combinations affect the final perturbation effect ranking as a whole. It can be seen that all the three strategies changed the ranking list with a similarity of ~0.6 on average. Also the combination strategy changed the ranking list mostly, which is expected.

## Discussion

In this study, we developed MUSIC, an integrated model-based pipeline designed specifically for single-cell CRISPR screening. MUSIC takes the raw counts data with the corresponding perturbation information as inputs and it can quantitatively estimate and prioritize the perturbation effect for each knockout or knockdown from three different perspectives, i.e., prioritizing the gene perturbation effect as an overall perturbation effect, in a functional topic-specific way, and quantifying the relationships between different perturbations. Extensive tests on MUSIC demonstrated that it is an effective and applicable pipeline for analyzing single-cell CRISPR screening data.

Single-cell CRISPR screening is a powerful technique, making it feasible to perform large-scale perturbations in a single-cell granularity. However, it is inherently noisy, presenting to be challenging for such data analysis. Currently version of MUSIC contains a series of carefully designed filtering steps to reduce the data noise, while future improvements are expected to refine and update such filtering steps to make it more effective.

## Methods

### Cell quality control

MUSIC evaluates cell quality based on three factors^29^, i.e., number of genes detected (default 500), number of unique molecular identifiers induced (default 1000), and percentage of mitochondrial genes detected (default 10% among all the detected genes). Only cells with the first two factors above the thresholds and the third factor below the threshold are retained.

### Data imputation

Single-cell RNA-seq data is sparse^10,11^, only a small fraction of the transcripts presented in each cell are sequenced. To improve the quality of data, MUSIC adopted SAVER^16^, a R package for single-cell RNA-seq data imputation which is proven to be necessary for MUSIC to discover the real and correct regulation relationships (Supplementary Table 2). It should be noted that SAVER has been proven to recover the true expression level of each gene in each individual cell, avoid to introduce spurious correlation or false positive gene pairs that have no biological correlations.

### Evaluation of sgRNA knockout efficiency

The sgRNA knockout efficiency in CRISPR screening should also be carefully evaluated. The sgRNA will target Cas9 to a specific gene locus, but only 70–80% of them will generate true loss-of-function of the targeted gene^30,31^. This implies that in 20–30% of the cells with a detected sgRNA, the gene can be active or partially active and show a wild-type phenotype (false positive) which will influence the estimation for the impact of perturbation. Thus, a step to filter such cells is needed. Intuitively, the basic idea of our filtering algorithm is based on the assumption that if the differentially expressed gene profile of a perturbed cell is more similar to the control cells than that of other same perturbed cells, this cell will be filtered. Specifically, for each type of perturbation, we performed the following steps:

- If the corresponding gene expression values of the perturbation are all zero among all the cells, this perturbation will be filtered directly. If not, perform the following steps.
- Identifying genes that are differentially expressed between control and perturbed cells by the Kolmogorov–Smirnov test at *p* < 0.05.
- For each perturbed cell *i*, the median of cosine similarity of differentially expressed gene profile between *i* and all the other perturbed cells with the same perturbation is calculated, denoted as *M*(_*Pi*_).
- For each perturbed cell *i*, the median of cosine similarity of differentially expressed gene profile between *i* and all the control cells is calculated, denoted as *M*(*C*_*i*_).
- For each cell *i*, if *M*(*C*_*i*_) is bigger than *M*(_*Pi*_), this cell will be filtered.
- For a specific perturbation, if the influenced cells filtered are amount to a high proportion (default 90%) among all, such perturbation is filtered.

### The minimal perturbed cell number per perturbation

Datlinger et al.^7^ concluded that at least 30 cells are required to capture each perturbation phenotype. Therefore, the perturbations with perturbed cells lower than 30 (default) are not considered in MUSIC.

### Selecting highly dispersion differentially expressed (DDE) genes

MUSIC identified differentially expressed genes in single-cell sequencing data as dispersion differentially expressed (DDE) genes, i.e., genes with a maximum dispersion difference (DD) between the case and control. MUSIC selects DDE genes based on the subsequent statistical test:

1 $${\mathrm{DD}}_i = \left| {{\mathrm{ZD}}_{{\mathrm{case}}}\left( i \right) - {\mathrm{ZD}}_{{\mathrm{control}}}\left( i \right)} \right|$$

where DD_*i*_ is the *i*-th gene’s dispersion difference, and ZD_case_(*i*) and ZD_control_(*i*) are the *z*-scores of the *i*-th gene’s dispersion in the case and control cells, respectively. Before calculating the *z*-score, the genes were binned based on their average expression, and the *z*-score of the dispersion was calculated within their corresponding bins. The *z*-score of the *i*-th gene’s dispersion (ZD_*i*_) is calculated as

2 $${\mathrm{ZD}}_i = \frac{{D_i - \mu _i}}{{\sigma _i}}$$

where *μ*_*i*_ and *σ*_*i*_ are the mean and variance of the *i*-th gene expression, respectively, within its corresponding bin and *D*_*i*_ is the dispersion of the *i*-th gene expression, which is calculated as

3 $$D_i = {\mathrm{log}}\frac{{\sigma _i}}{{\mu _i}}$$

where *σ*_*i*_ and *μ*_*i*_ are the variance and mean, respectively, of the *i*-th gene expression.

### Normalizing and rounding the expression value

The expression level of different genes is normalized and rounded to fit the topic model:

4 $$X_{{\mathrm{normalized}}} = \left[ {\frac{{X - \mu _{{\mathrm{control}}}}}{{\mu _{{\mathrm{control}}}}} \times 10} \right]$$

We round the final expression value as the ×10 magnification of the original normalized expression values.

### Topic models

The topic model was originally presented in the machine-learning and natural language processing community for latent topics discovery in a particular set of documents^17^. This generative hierarchical model assumes that a word in a document is generated through two steps, i.e., a topic in a document is selected with a certain probability, and then a word in the topic is selected with a certain probability. The generative process of topic model is formulated as follows: *θ*_*d*_ and $\O _t$ are, respectively, the distribution over topics of document *d* and the distribution over words of topic *t*.

5 $$\theta _t{\mathrm{\sim Dirichlet}}\left( \alpha \right)$$

6 $$∅ _t{\mathrm{\sim Dirichlet}}\left( \beta \right)$$

Here, *α* and *β* are hyper-parameters following Dirichlet distributions. For generating word *i* in document *d*, topic *Z*_*d*,*i*_ is first sampled from document’s distribution over topics, and then word *W*_*d*,*i*_ is sampled from the topic’s distribution over words based on the following distributions,

7 $$Z_{d,i} \vee \theta _d{\mathrm{\sim Multinomial}}\left( {\theta _d} \right)$$

8 $$W_{d,i} \vee Z_{d,i},∅ _{Z_{d,i}}{\mathrm{\sim Multinomial}}\left( {Z_{d,i}} \right)$$

In our study, the topic model is utilized to process our single-cell CRISPR screening data. We made a perfect analogy between text mining and perturbation effect evaluation, where documents can be analogized to the cells conducted by single-cell CRISPR screening and the word frequency in a document can be analogized to the expression value of genes for a given cell. We determined the joint probability of gene expression for each cell by integrating parameter *θ* into ∅ and applied the collapsed Gibbs sampling to assign the gene of each cell to topics. Detailed information can be refereed^17^.

In summary, topic modeling was performed on the entire screen dataset to compare the impact of different perturbations under the same background. Topic modeling resulted into two outputs, i.e., (1) the probability distribution of each topic, representing as a topic profile, which is used to characterize each perturbation (include control) and (2) the enriched functional profile of each topic, which is intuitively calculated by the enrichment analysis with top 10% differentially expressed genes in each topic. Then, with such two profiles in hand, we are able to quantitatively calculate the overall perturbation effect ranking, topic-specific perturbation ranking as well as the relationship between perturbations.

### Annotating each topic’s function

MUSIC obtains the occurrence probabilities of genes available in each topic. For each topic, MUSIC took full advantage of the power of topic profile modeling to perform a weighted biological function annotation. Intuitively, genes with large occurrence probabilities are more representative of the function and they should be selected to annotate the topic function. Specifically, for each topic, MUSIC performed the following steps:

- MUSIC first selects the top 10% genes of each topic based on their occurrence probabilities.
- Genes selected by step 1 are used to perform the functional enrichment annotation with clusterProfiler^32^.
- In the end, the top-ranked *n* (default 5) GO terms (rank by *q* value) are selected to represent the topic functions.

### Automatically selecting the optimal topic number

Topic distribution is influenced by the topic number. MUSIC applies an automatic strategy to select the optimal topic number. Intuitively, an optimal topic number should distinguish the cells with different perturbation effects from each other as much as possible. In our study, we defined a matrix *G*_*m*×*n*_ representing the *n* topics’ occurrence probability in *m* cells derived from the topic model with a certain topic number *n*. Then, an optimal topic number should make *G*_*m*×*n*_ match the following two criteria: (I) For each topic, its occurrence probability in different perturbation cells should differ as much as possible. Such a measurement is defined as a specificity score (SS_*n*_) for all the topics under a certain topic number *n*, as calculated in Eq. (9). The larger the specificity score, the better the selected topic number. (II) The fewer topic functions dominating each cell, the better. Such a measurement is defined as a purity score (PS_*n*_) for all the topics under certain topic number *n*, as calculated in Eq. (10). The larger the score, the better the selected topic number. Finally, MUSIC defined the combination score(CS_*n*_), which is a weighted average of the specificity score and purity score, as shown in Eq. (11). Again, the larger the score, the better the selected topic number.

The specificity score (SS_*n*_) is calculated as

9 $${\mathrm{SS}}_n = {\mathrm{log}}\left( {\frac{1}{n}\mathop {\sum }\limits_{j = 1}^n \frac{{\sigma _j}}{{\mu _j^2}}} \right)$$

where *n* is the selected topic number, and *σ*_*j*_ and *μ*_*j*_ are the variance and mean, respectively, of the *j*-th column of *G*_*m*×*n*_.

The purity score (PS_*n*_) is calculated as

10 $${\mathrm{PS}}_n = {\mathrm{log}}\frac{1}{m}\mathop {\sum }\limits_{i = 1}^m \sigma _i$$

where *n* is the selected topic number, *m* is the number of rows in matrix *G*_*m*×*n*_, and *σ*_*i*_ is the variance of the *i*-th row of *G*_*m*×*n*_.

The combination score (CS_*n*_) is calculated as

11 $${\mathrm{CS}}_n = \alpha {\mathrm{TSS}}_n + \left( {1 - \alpha } \right){\mathrm{TPS}}_n$$

where *n* is the selected topic number and *α* (default 0.5) is the weight with value of [0, 1]. Considering the time cost and the biological interpretability of the result, we recommended a reasonable scope (now 4 to 6) of topic model number to be tried, by considering the prior information of biologic functional categories.

### Considering off-target effects

A sgRNA off-target effect may exist for these novel types of data due to application of the CRISPR knockout/knockdown screening technique. For CRISPRi technique, MUSIC won’t consider this step, since CRISPRi knockdown is highly specific with minimal off-target effects^33^. In the current version MUSIC only provides the off-target information of the knockout. Basically, MUSIC integrates sgRNA sequence information with its corresponding knockout gene expression to determine whether the sgRNA has induced an off-target effect as following:

- CRISPRseek^34^ is performed to predict possible off-targets based on the sgRNA sequence information.
- Correlations of the transcriptional expression values between the corresponding knockout gene and the possible off-targets are calculated for the case and control, respectively.
- If a significant increase in the correlations between the case and control is detected, the possible off-target effect for this knockout is reported in MUSIC.

### Obtaining the topic-specific ranking list

To analyze the functions of the perturbations impact, MUSIC prioritizes the perturbation effect in a topic-specific way. For a specific topic, MUSIC prioritizes the perturbation effect by calculating the specific topic probability difference (TPD) between the case and control. Intuitively, the ranking list is obtained by evaluating the perturbation effect on this specific topic, where the perturbation should influence this topic as much as possible while keeping other topics as unaffected as possible. Specifically, MUSIC performed the following steps:

1. MUSIC calculates topic probability difference (TPD) based on Student *t*-test. In order to meet the conditions of the Student *t*-test, the topic probability of different cells with different perturbation were normalized to the standard normal distribution. Specifically, for the *i*-th perturbation on the *j*-th topic, each topic probability was *z*-normalized with respect to the mean and standard deviation of the corresponding control population as:
  12 $$P_{{\mathrm{normalized}}}\left( {i,j} \right) = \frac{{P\left( {i,j} \right) - \mu _{{\mathrm{control}}}}}{{\sigma _{{\mathrm{control}}}}}$$
2. We also realized that the number of cells with different edits generally varies greatly, i.e., the sample imbalance issue exists, which can affect the analysis of the perturbation effects greatly. To address this issue, MUSIC first identified the minimal cell number (*M*) among all perturbations. Then, for each perturbation, MUSIC adopted a bootstrapping strategy to randomly samples *M* cells to perform the subsequent Student *t*-test for 1000 times, and the median is obtained. The test statistic of the *i*-th perturbation on the *j*-th topic is calculated as
  13 $${\mathrm{TPD}}_{ij} = \frac{{\bar X_{ij} - \bar X_{{\mathrm{control}},j}}}{{\sqrt {\left( {\frac{{\left( {n_i - 1} \right)S_{ij}^2 + \left( {n_{{\mathrm{control}}} - 1} \right)S_{{\mathrm{control}},j}^2}}{{n_i + n_{{\mathrm{control}}} - 2}}\left( {\frac{1}{{n_i}} + \frac{1}{{n_{{\mathrm{control}}}}}} \right)} \right)} }}$$
  where $\bar X_{ij}$ is the mean of normalized topic probabilities calculated in Eq. (12) for the *i*-th perturbation on the *j*-th topic, $\bar X_{{\mathrm{control}},j}$ is the mean of normalized topic probabilities of control cells for the *j*-th topic, *S*_*ij*_ is the standard deviation of normalized topic probabilities of cells for the *i*-th perturbation on the *j*-th topic, *S*_control,*j*_ is the standard deviation of normalized topic probabilities of control cells for the *j*-th topic.
  In our study, the test statistic TPD will be taken for consideration for the following two reasons: (a) TPD is a valid metric to estimate the difference of mean between two populations. (b) TPD can be positive or negative, thus used to estimate the direction of a perturbation impact.
3. Then, MUSIC prioritizes such a perturbation by considering the effect of the perturbation on this specific topic as well as its influence on other topics.
  MUSIC applies the ratio of each topic probability difference (TPDR) to evaluate its influence on other topics. The bigger the ratio is, the less the perturbation influence on other topics.
  The TPDR of the *i*-th perturbation on the *j*-th topic is calculated as
  14 $${\mathrm{TPDR}}_{ij} = \frac{{\left| {{\mathrm{TPD}}_{ij}} \right|}}{{\mathop {\sum }\nolimits_{i = 1}^n \left| {{\mathrm{TPD}}_{ij}} \right|}}$$
  where TPD_*ij*_ is calculated in Eq. (13).
4. Finally, MUSIC defines an efficient score to evaluate the effect of the *i*-th perturbation (CS_*i*_) on a specific topic considering both TPD and TPDR. The larger the score, the higher the rank.
  15 $${\mathrm{CS}}_{ij} = 0.5 \ast \left( \frac{\left. \left| {\mathrm{TPD}}_{ij} \right| - \min \left( \left| {\mathrm{TPD}}_{i.} \right| \right) \right)}{\max \left( \left| {\mathrm{TPD}}_{i.} \right| \right) - \min \left( \left| {\mathrm{TPD}}_{i.} \right| \right)} + \frac{{\mathrm{TPDR}}_{ij} - {\mathrm{min}}({\mathrm{TPDR}}_{ij})}{\max \left( {\mathrm{TPDR}}_{ij} \right) - {\mathrm{min}}({\mathrm{TPDR}}_{ij})} \right)$$

MUSIC also calculated a threshold to determine if a perturbation had an impact on a specific topic with statistically significance. Intuitively, the impact of a perturbation on a functional topic is significant if it is greater than that generated randomly. MUSIC first obtained TPD_random,*j*_ which can be calculated in Eq. (16) and performs the same process to obtain the score (CS) between selected ones and all. This process is repeated for 1000 times to obtain the median as the threshold. The impact of the *i*-th perturbation on a specific topic *j* is considered significant when CS_*ij*_ is bigger than the threshold.

16 $${\mathrm{TPD}}_{{\mathrm{random}},j} = \frac{{\bar X_{{\mathrm{random}},j} - \bar X_{{\mathrm{control}},j}}}{{\sqrt {\left( {\frac{{\left( {n_{{\mathrm{random}}} - 1} \right)S_{{\mathrm{random}},j}^2 + \left( {n_{{\mathrm{control}}} - 1} \right)S_{{\mathrm{control}},j}^2}}{{n_{{\mathrm{random}}} + n_{{\mathrm{control}}} - 2}}\left( {\frac{1}{{n_{{\mathrm{random}}}}} + \frac{1}{{n_{{\mathrm{control}}}}}} \right)} \right)} }}$$

where $\bar X_{{\mathrm{random}},j}$ is the mean of normalized topic probabilities calculated in Eq. (12) for the *M* selected control cells on the *j*-th topic.

### Obtaining the overall perturbation effect ranking list

For the calculation of the overall perturbation effect ranking list, the sum of each topic’s TPD (TPDS) for each perturbation was calculated:

17 $${\mathrm{TPDS}}_i = \mathop {\sum }\limits_{j = 1}^n \left| {{\mathrm{TPD}}_{ij}} \right|$$

It should be noted that in practical the calculation of TPD here is needed to be adjusted by performing the same bootstrapping on control cells. Specifically, the adjust TPD, i.e., TPDA is calculated as

18 $${\mathrm{TPDA}}_{ij} = {\mathrm{TPD}}_{ij} - {\mathrm{TPD}}_{{\mathrm{random}},j}$$

### Obtaining the relationships between different perturbations

MUSIC quantifies the relationships between two perturbations by calculating the Pearson correlation coefficient of two perturbations’ TPDA profiles. Furthermore, the perturbation correlation networks can be automatically visualized by MUSIC for each testing dataset, respectively.

### Prioritizing perturbation effect difference under different treatment conditions

When cells were treated under different experimental conditions, MUSIC can be applied to prioritize the perturbation effect difference under two different conditions, and identify the perturbation with substantial effect change. Intuitively, by comparing the TPDS of one specific perturbation under two different conditions, MUSIC identified those perturbations whose impact changed significantly under two conditions. Specifically, MUSIC first selected the common perturbations under two conditions, then MUSIC defined the score perturbation impact difference (PID) to quantitatively represent the perturbation impact difference between two different experimental conditions. For a perturbation i, PID_*i*_ is calculated as

19 $${\mathrm{PID}}_i = \frac{{{\mathrm{TPDS}}({\mathrm{condition}}\_2)_i}}{{\mathop {\sum }\nolimits_i^n {\mathrm{TPDS}}({\mathrm{condition}}\_2)_i}}/\frac{{{\mathrm{TPDS}}({\mathrm{condition}}\_1)_i}}{{\mathop {\sum }\nolimits_i^n {\mathrm{TPDS}}({\mathrm{condition}}\_1)_i}}$$

where *n* is the number of common perturbations under two conditions and TPDS is calculated by Eq. (17).

### Comparisons between negative control and blank control

Given that the former steps rely on the comparisons between perturbed and negative control cells, we made a statistical test to compare negative control with blank control to indicate the suitability of applying negative control in the experiments.

First, we believe that it should be slightly different to use the negative control (induced with non-targeting gRNAs) and the blank control (none gRNAs induced) in the single-cell CRISPR screening experiments. While in the previous studies^4–8^, researchers in this community tend to choose negative control rather than blank control to keep a relative fair comparison scenario, since it is necessary to eliminate the effects of the induction on the cells.

Second, the differences between negative control and blank control should be less significantly than that between knockouts/knockdowns and blank control. To prove this point, we made the following test with stimulated Jurkat cell^7^ which offered cells without any induction of gRNAs (blank control). The routing imputation and filtering were performed on these cells. Then a bootstrap sampling strategy is applied on the blank control cells to randomly selected 10% among them to compare with negative control and other knockouts cells. Then we calculated the similarity of such comparison for 100 times samplings. The statistical comparison result is shown in Supplementary Fig. 15. It is clearly to see that the negative control cells are significantly similar to blank control (*t*-test *p* < 2.2e−16) than any other knockouts.

### Robust test

For each datasets, we randomly relabeled 20% control cells as a control test subset to be processed along other knockouts or knockdowns, and calculated the rank of the control test subset in the overall perturbation effect ranking result. We calculated the rate of the knockouts or knockdowns whose rank below the control test subset among the total number of knockouts or knockdowns. The above process was repeated 10 times for each datasets to reduce randomness. The average rate calculated above is about 0.06 among all the available datasets, indicating that the control testing sets in general disturb the final ranking list a little. Besides, for each datasets, the Pearson correlation coefficients were similarly calculated as aforementioned between the overall perturbation effect ranking results obtained from this random test and that from the original studies. The average Pearson correlation coefficient is 0.82, further indicating that the data preprocessing steps in MUSIC is reliable and robust with tolerance to the random noise.

### Reporting summary

Further information on research design is available in the Nature Research Reporting Summary linked to this article.

## Supplementary information

Supplemetary Information

Reporting Summary

Description of Additional Supplementary Files

Supplementary Data 1

Supplementary Data 2

Supplementary Data 3

Supplementary Data 4

Supplementary Data 5

Supplementary Data 6

Supplementary Data 7

Supplementary Data 8

Supplementary Data 9

Supplementary Data 10

Supplementary Data 11

Supplementary Data 12

Supplementary Data 13

Supplementary Data 14

Supplementary Data 15

Supplementary Data 16

Supplementary Data 17

## Data Availability

The datasets analyzed during the current study are available in the Gene Expression Omnibus (GEO) repository with the accession codes: GSE90063, GSE90546, GSE90486, GSE92872, GSE108699. All other relevant data are available upon request.

## Code availability

MUSIC is available as an R package at https://github.com/bm2-lab/MUSIC with a Docker version for a quick deployment at https://hub.docker.com/r/bm2lab/music/.

## References

[1] Wang T, Wei JJ, Sabatini DM, Lander ES (2014). Genetic screens in human cells using the CRISPR-Cas9 system. Science.

[2] Shalem O (2014). Genome-scale CRISPR-Cas9 knockout screening in human cells. Science.

[3] Lanning BR, Vakoc CR (2017). Single-minded CRISPR screening. Nat. Biotechnol..

[4] Adamson B (2016). A multiplexed single-cell CRISPR screening platform enables systematic dissection of the unfolded protein response. Cell.

[5] Dixit A (2016). Perturb-seq: dissecting molecular circuits with scalable single-cell RNA profiling of pooled genetic screens. Cell.

[6] Jaitin DA (2016). Dissecting immune circuits by linking CRISPR-pooled screens with single-cell RNA-Seq. Cell.

[7] Datlinger P (2017). Pooled CRISPR screening with single-cell transcriptome readout. Nat. Methods.

[8] Hill AJ (2018). On the design of CRISPR-based single-cell molecular screens. Nat. Methods.

[9] Junker JP, van Oudenaarden A (2014). Every cell is special: genome-wide studies add a new dimension to single-cell biology. Cell.

[10] Pierson E, Yau C (2015). ZIFA: dimensionality reduction for zero-inflated single-cell gene expression analysis. Genome Biol..

[11] Lun AT, Bach K, Marioni JC (2016). Pooling across cells to normalize single-cell RNA sequencing data with many zero counts. Genome Biol..

[12] Brennecke P (2013). Accounting for technical noise in single-cell RNA-seq experiments. Nat. Methods.

[13] Fu Y (2013). High-frequency off-target mutagenesis induced by CRISPR-Cas nucleases in human cells. Nat. Biotechnol..

[14] Tsai SQ (2015). GUIDE-seq enables genome-wide profiling of off-target cleavage by CRISPR-Cas nucleases. Nat. Biotechnol..

[15] Tsai SQ (2017). CIRCLE-seq: a highly sensitive in vitro screen for genome-wide CRISPR-Cas9 nuclease off-targets. Nat. Methods.

[16] Huang M (2018). SAVER: gene expression recovery for single-cell RNA sequencing. Nat. Methods.

[17] Blei DM, Lafferty JD (2007). A correlated topic model of science. Ann. Appl Stat..

[18] Huang Y, Gilna P, Li W (2009). Identification of ribosomal RNA genes in metagenomic fragments. Bioinformatics.

[19] Yan J (2017). MetaTopics: an integration tool to analyze microbial community profile by topic model. BMC Genom..

[20] Dey KK, Hsiao CJ, Stephens M (2017). Visualizing the structure of RNA-seq expression data using grade of membership models. PLoS. Genet..

[21] Kinoshita S, Akira S, Kishimoto T (1992). A member of the C/EBP family, NF-IL6 beta, forms a heterodimer and transcriptionally synergizes with NF-IL6. Proc. Natl Acad. Sci. USA.

[22] Rorth P, Szabo K, Texido G (2000). The level of C/EBP protein is critical for cell migration during *Drosophila* oogenesis and is tightly controlled by regulated degradation. Mol. Cell.

[23] Liu Y (2017). beta-elemene regulates endoplasmic reticulum stress to induce the apoptosis of NSCLC cells through PERK/IRE1alpha/ATF6 pathway. Biomed. Pharmacother..

[24] Huber R, Pietsch D, Panterodt T, Brand K (2012). Regulation of C/EBPbeta and resulting functions in cells of the monocytic lineage. Cell. Signal..

[25] Weber M (2003). Transcriptional inhibition of interleukin-8 expression in tumor necrosis factor-tolerant cells: evidence for involvement of C/EBP beta. J. Biol. Chem..

[26] Aas T (1996). Specific P53 mutations are associated with de novo resistance to doxorubicin in breast cancer patients. Nat. Med..

[27] Vikhanskaya F, D’Incalci M, Broggini M (1995). Decreased cytotoxic effects of doxorubicin in a human ovarian cancer-cell line expressing wild-type p53 and WAF1/CIP1 genes. Int. J. Cancer.

[28] Hochhauser D (1999). Effects of wild-type p53 expression on the quantity and activity of topoisomerase IIalpha and beta in various human cancer cell lines. J. Cell. Biochem..

[29] Satija R, Farrell JA, Gennert D, Schier AF, Regev A (2015). Spatial reconstruction of single-cell gene expression data. Nat. Biotechnol..

[30] Lappalainen T (2013). Transcriptome and genome sequencing uncovers functional variation in humans. Nature.

[31] Popp MW, Maquat LE (2016). Leveraging rules of nonsense-mediated mRNA Decay for genome engineering and personalized medicine. Cell.

[32] Yu G, Wang LG, Han Y, He QY (2012). clusterProfiler: an R package for comparing biological themes among gene clusters. OMICS.

[33] Gilbert LA (2013). CRISPR-mediated modular RNA-guided regulation of transcription in eukaryotes. Cell.

[34] Zhu LJ, Holmes BR, Aronin N, Brodsky MH (2014). CRISPRseek: a bioconductor package to identify target-specific guide RNAs for CRISPR-Cas9 genome-editing systems. PLoS. One..

[35] Nuchprayoon I, Simkevich CP, Luo M, Friedman AD, Rosmarin AG (1997). GABP cooperates with c-Myb and C/EBP to activate the neutrophil elastase promoter. Blood.

[36] Odrowaz Z, Sharrocks AD (2012). The ETS transcription factors ELK1 and GABPA regulate different gene networks to control MCF10A breast epithelial cell migration. PLoS. One..

[37] Liu K, Lin FT, Graves JD, Lee YJ, Lin WC (2017). Mutant p53 perturbs DNA replication checkpoint control through TopBP1 and Treslin. Proc. Natl Acad. Sci. USA.

