# Supplementary material for: Model-based understanding of single-cell CRISPR screening
Source: Nat Commun. 2019 May 20;10:2233. doi: 10.1038/s41467-019-10216-x (PMC6527552; doi:10.1038/s41467-019-10216-x)
Supplement: Supplementary file 1 — Supplemetary Information [file 41467_2019_10216_MOESM1_ESM.pdf]

Supplementary Information for

Model-based Understanding of Single-cell CRISPR screening

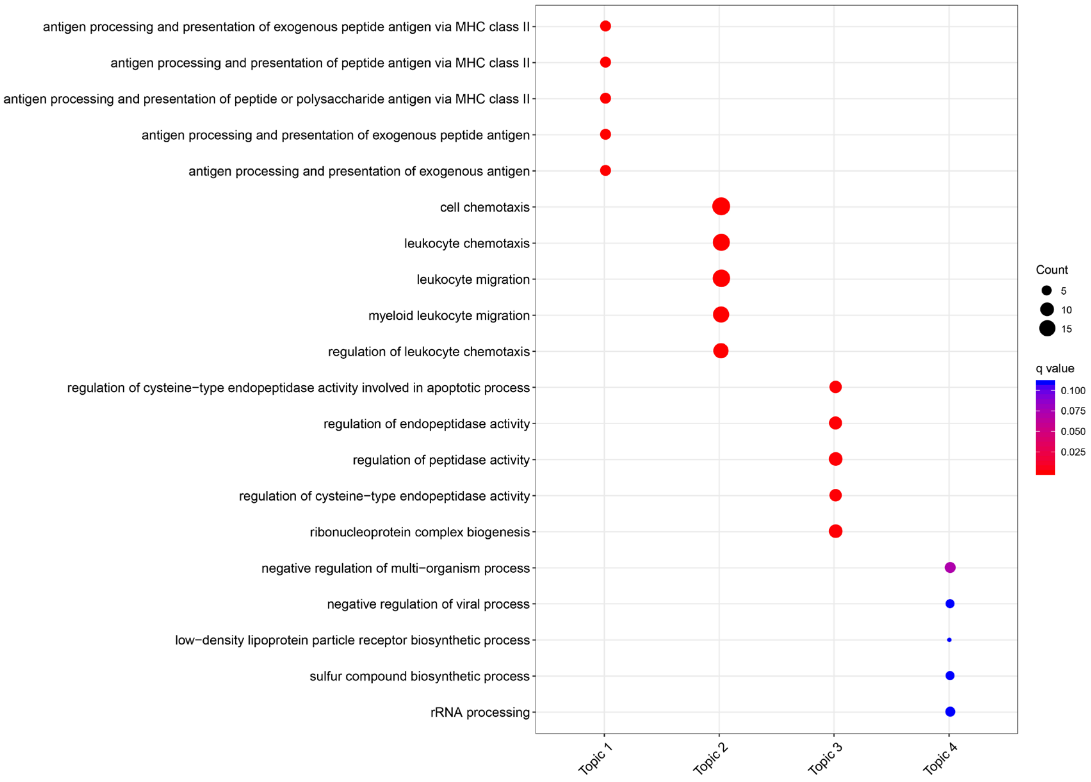

Supplementary Figure 1 Functional annotation of topics for stimulated BMDC (GSM2396856).

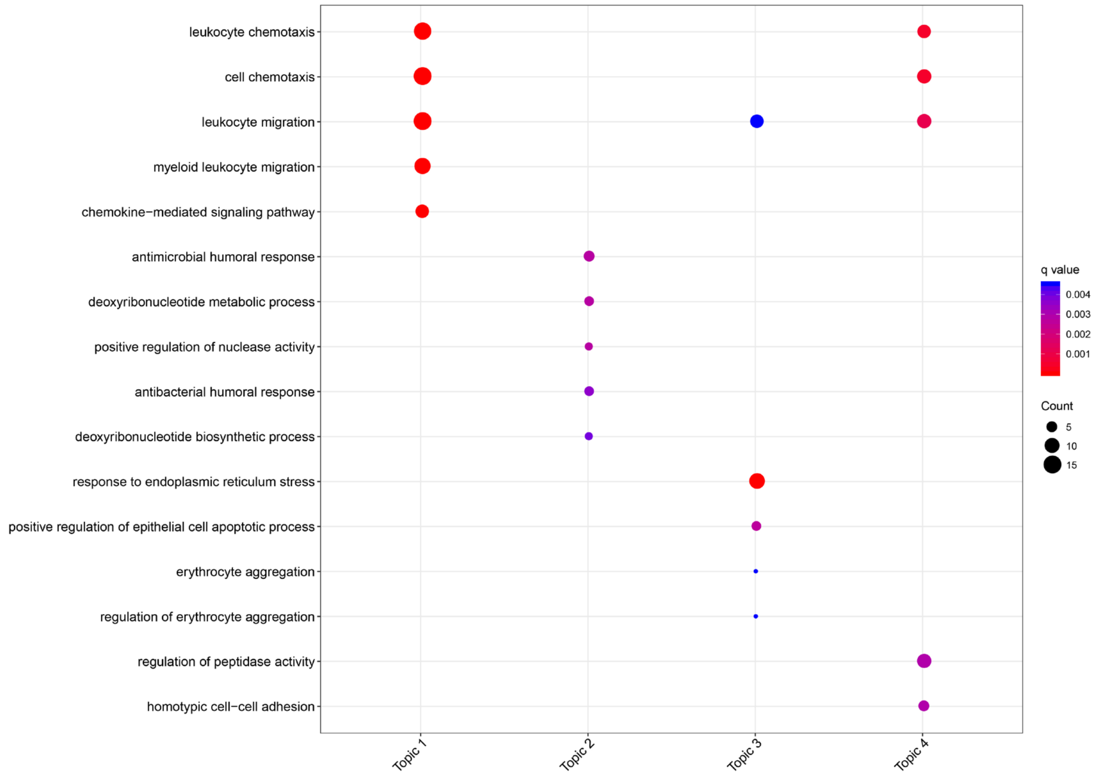

Supplementary Figure 2 Functional annotation of topics for unstimulated BMDC (GSM2396857).

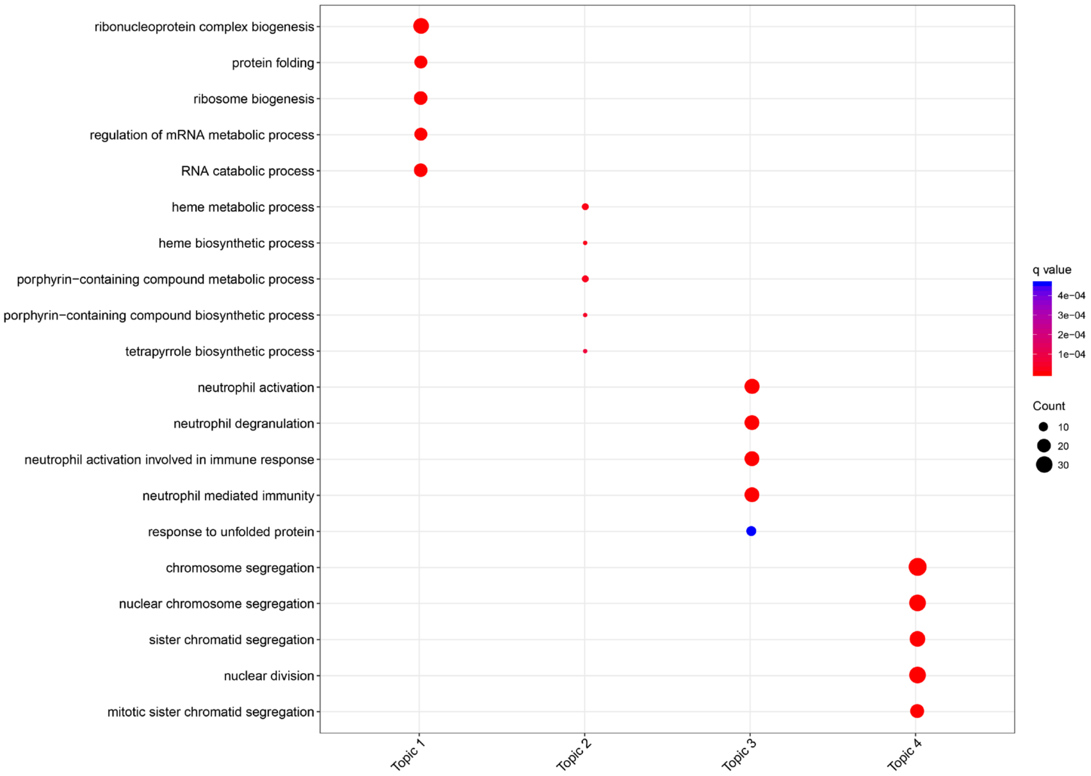

Supplementary Figure 3 Functional annotation of topics for K562 with 7 days post transduction (GSM2396858).

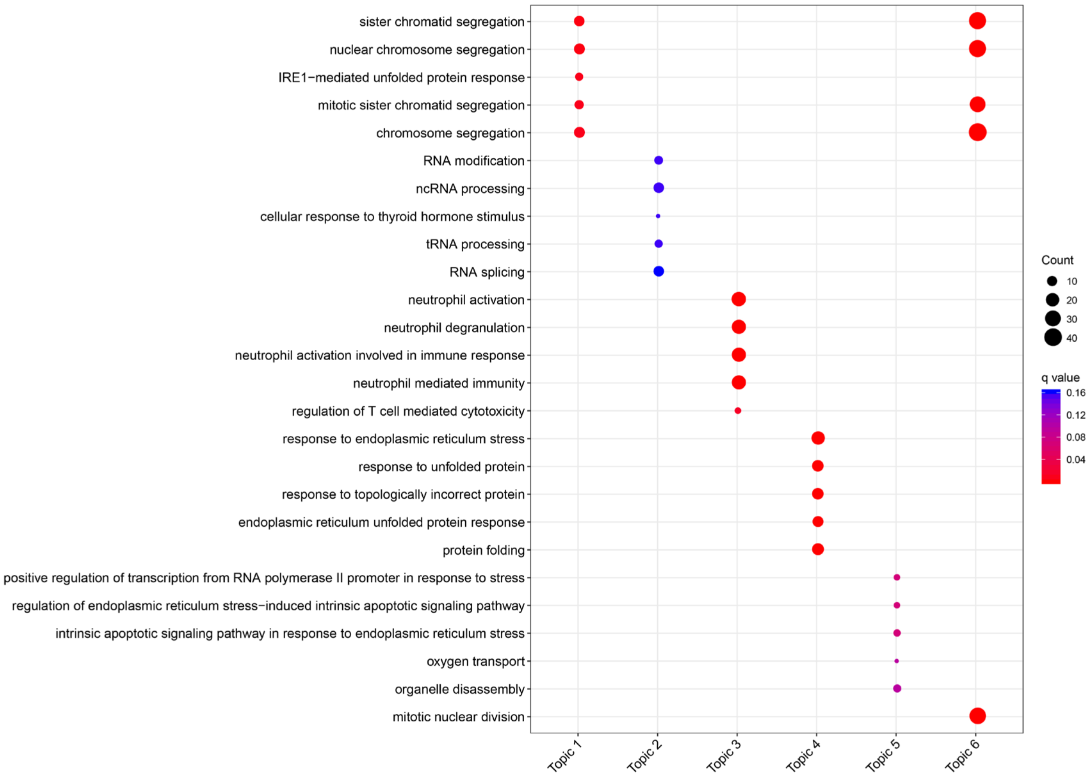

Supplementary Figure 4 Functional annotation of topics for K562 with 13 days post transduction (GSM2396859).

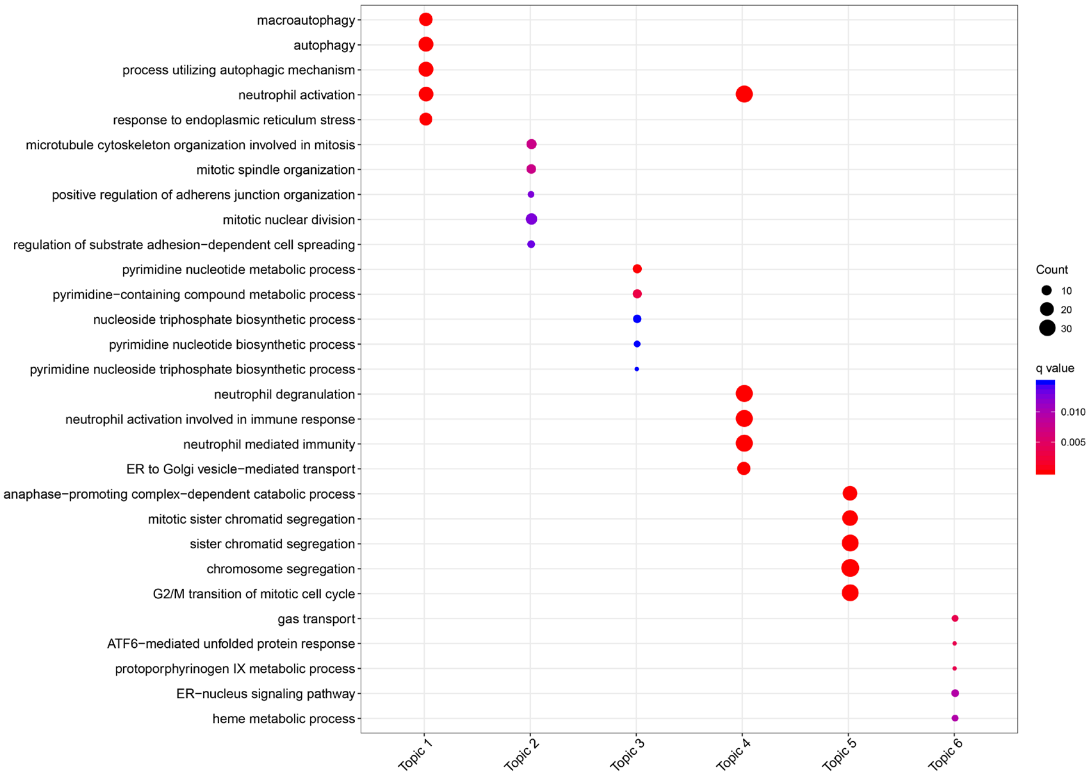

Supplementary Figure 5 Functional annotation of topics for K562 with high MOI (GSM2396860).

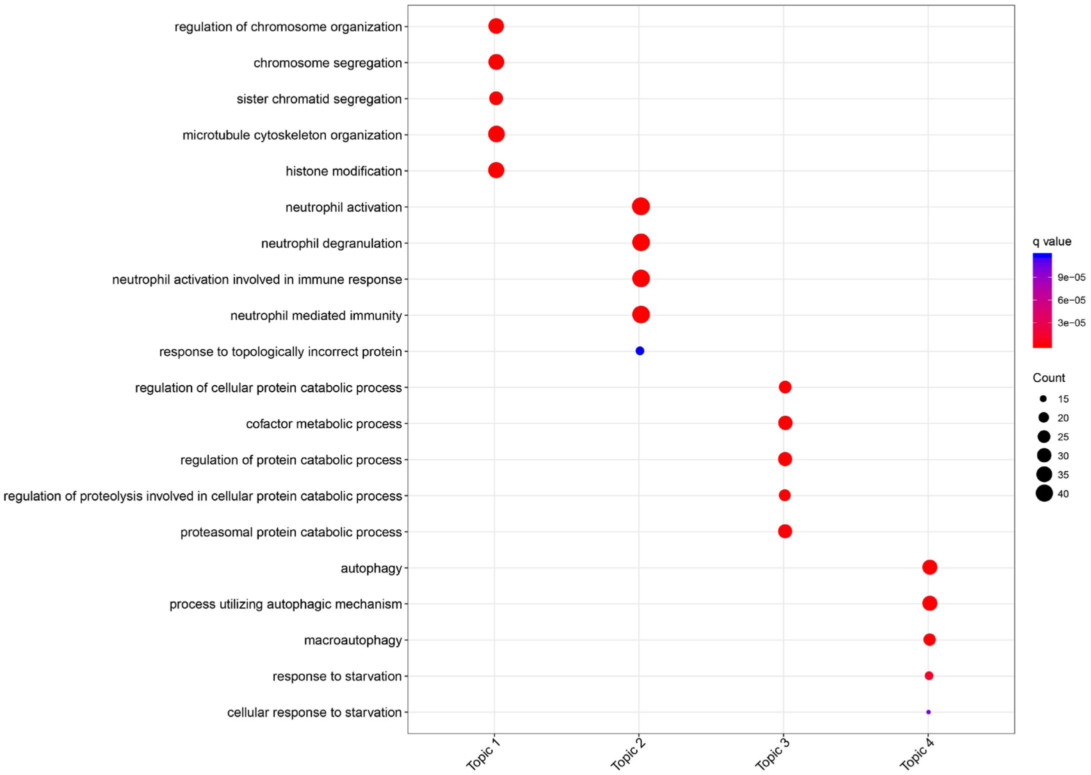

Supplementary Figure 6 Functional annotation of topics for K562 with perturbation of cell cycle regulators (GSM2396861).

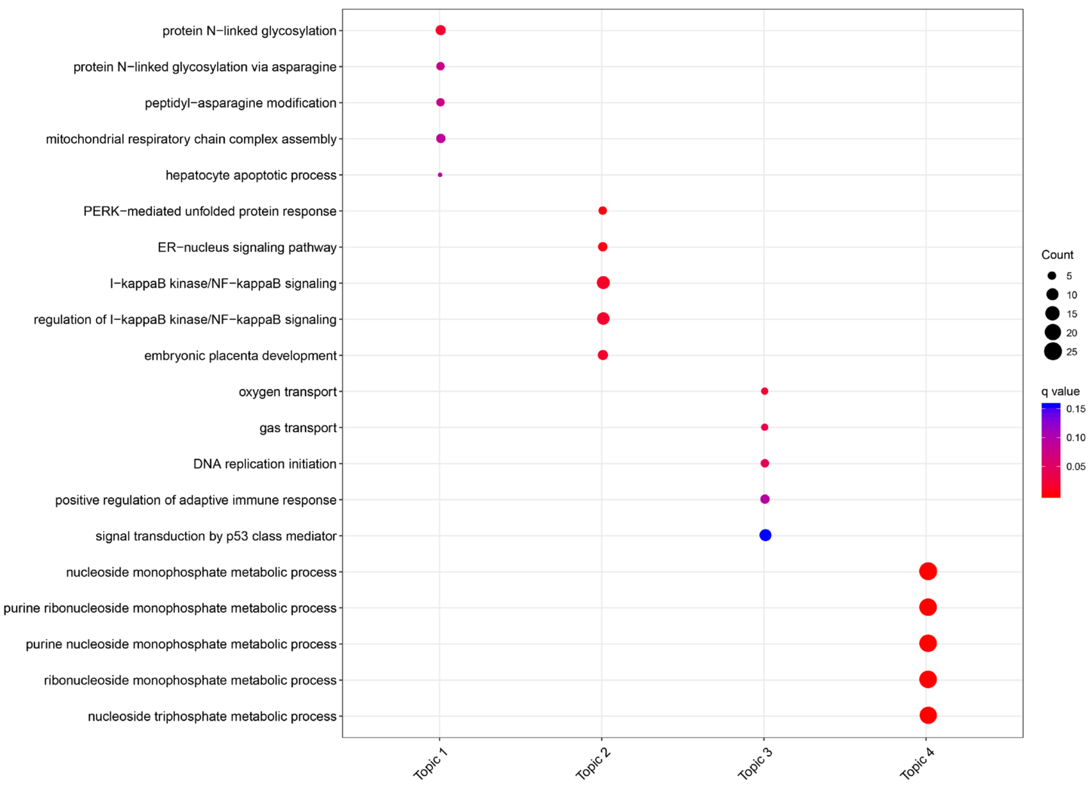

Supplementary Figure 7 Functional annotation of topics for K562 cells (GSM2406675).

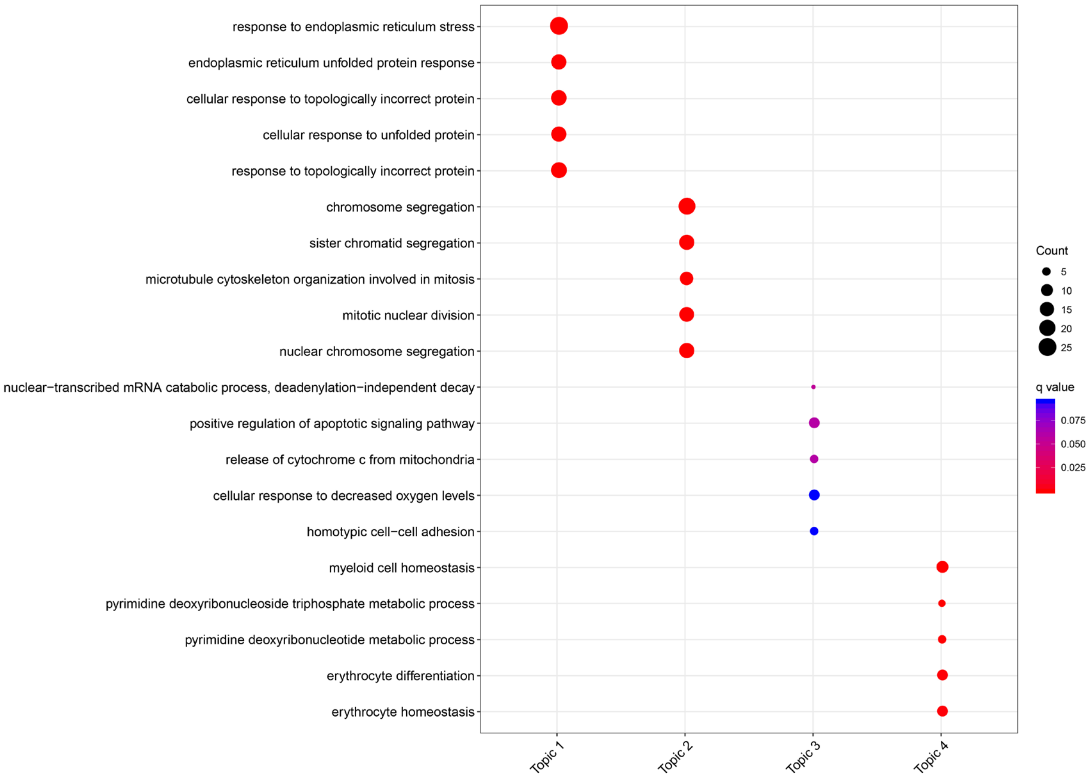

Supplementary Figure 8 Functional annotation of topics for K562 with three UPR genes (GSM2406677).

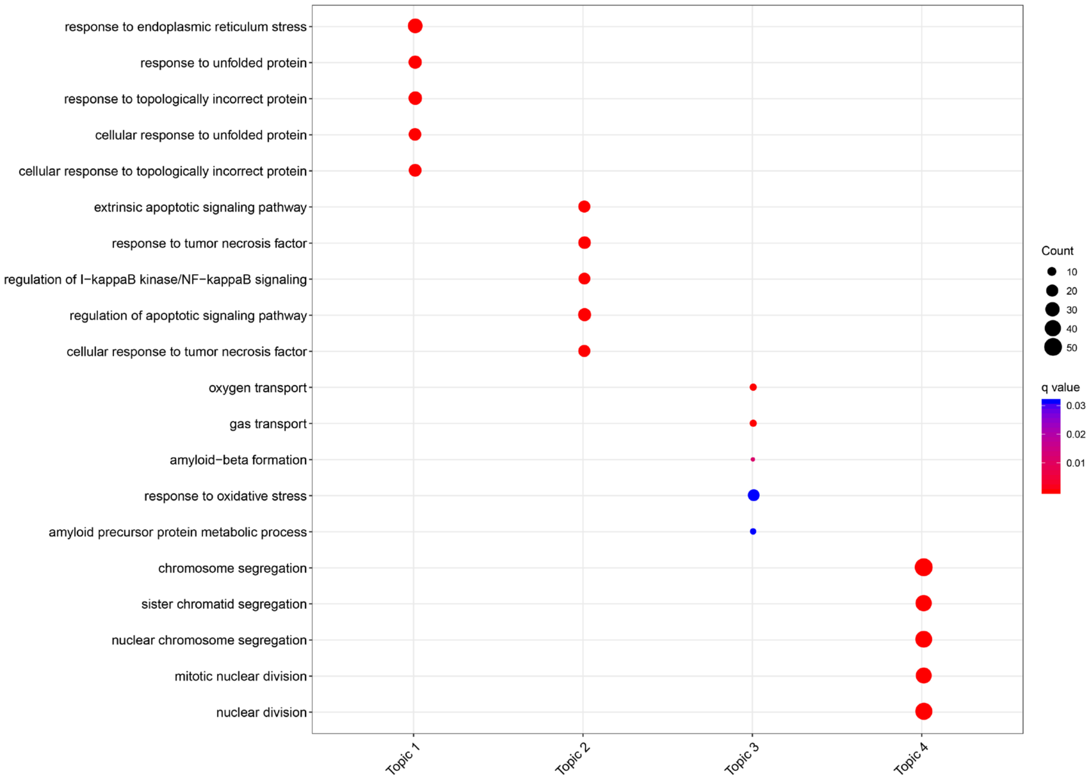

Supplementary Figure 9 Functional annotation of topics for K562 with the perturbation of 83 UPR genes (GSM2406681).

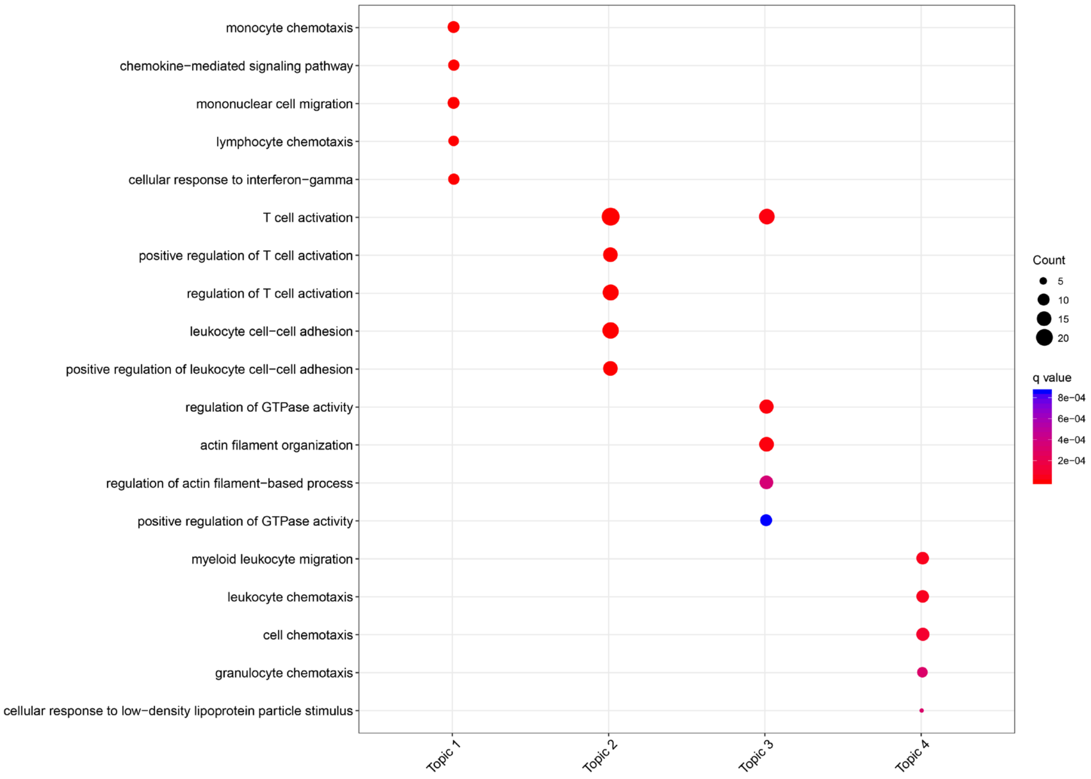

Supplementary Figure 10 Functional annotation of topics for myeloid cells (GSE90486).

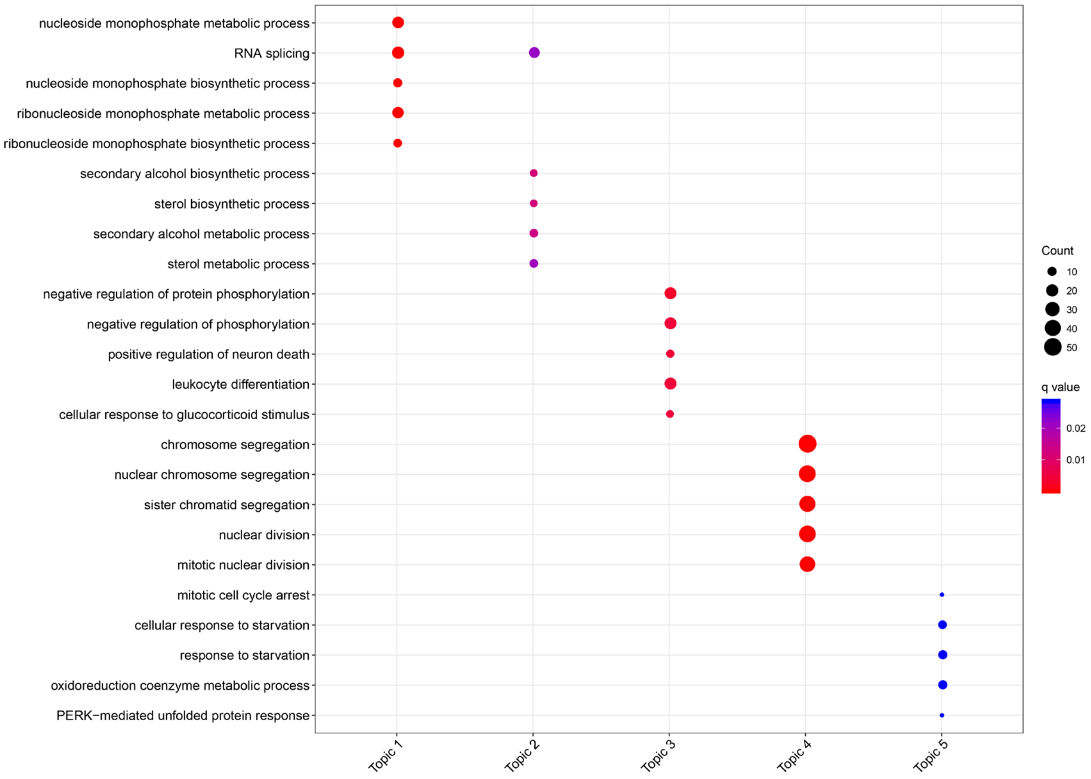

Supplementary Figure 11 Functional annotation of topics for anti CD3/CD28 stimulated Jurkat cells (GSM2439080 ~ GSM2439085).

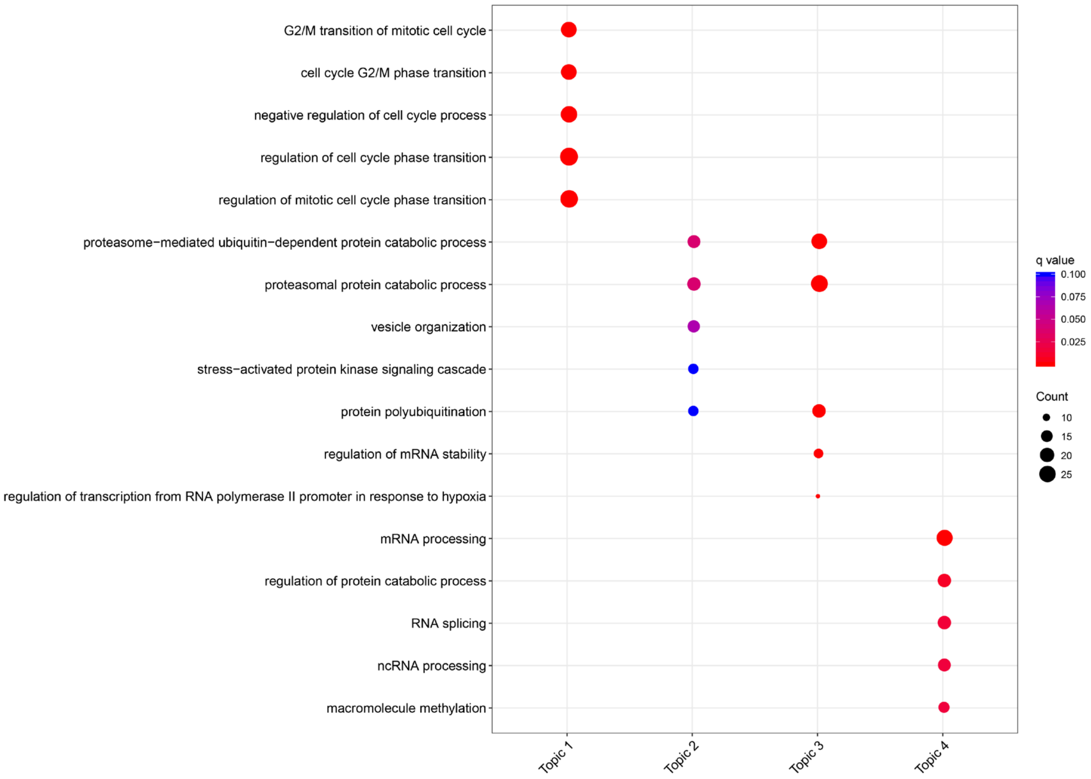

Supplementary Figure 12 Functional annotation of topics for unstimulated Jurkat cells (GSM2439086 ~ GSM2439090).

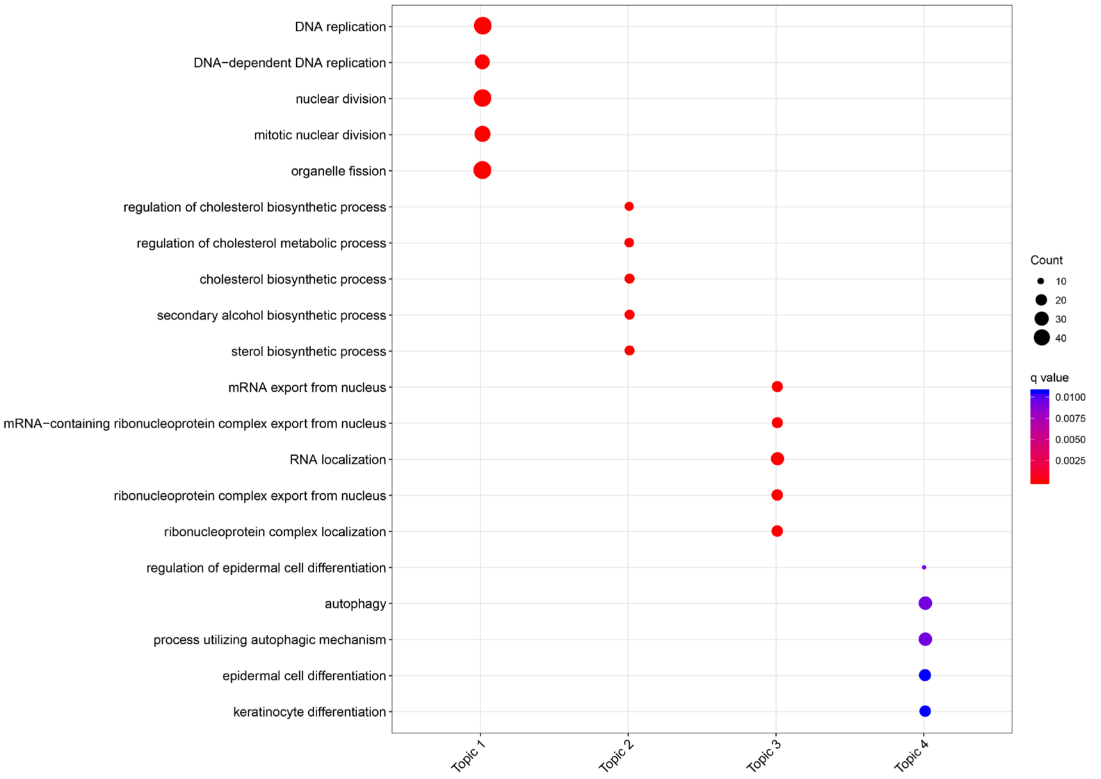

Supplementary Figure 13 Functional annotation of topics for doxorubicin treated MCF10A (GSM2911346).

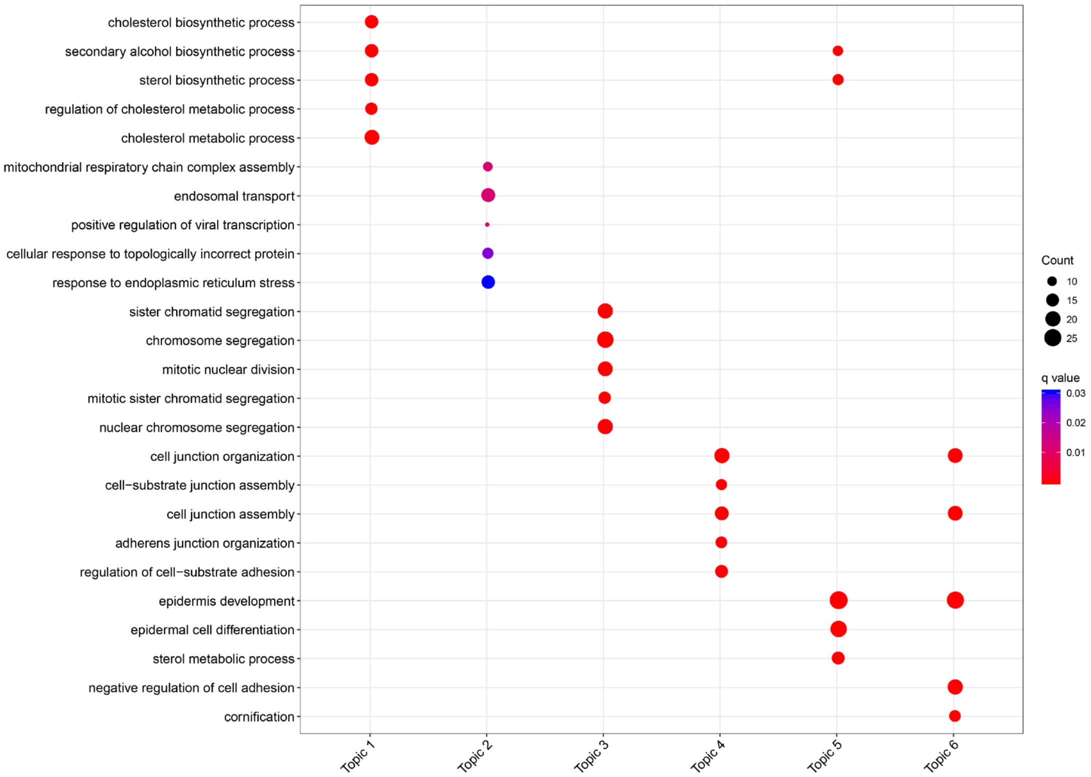

Supplementary Figure 14 Functional annotation of topics for untreated MCF10A cells (GSM2911347).

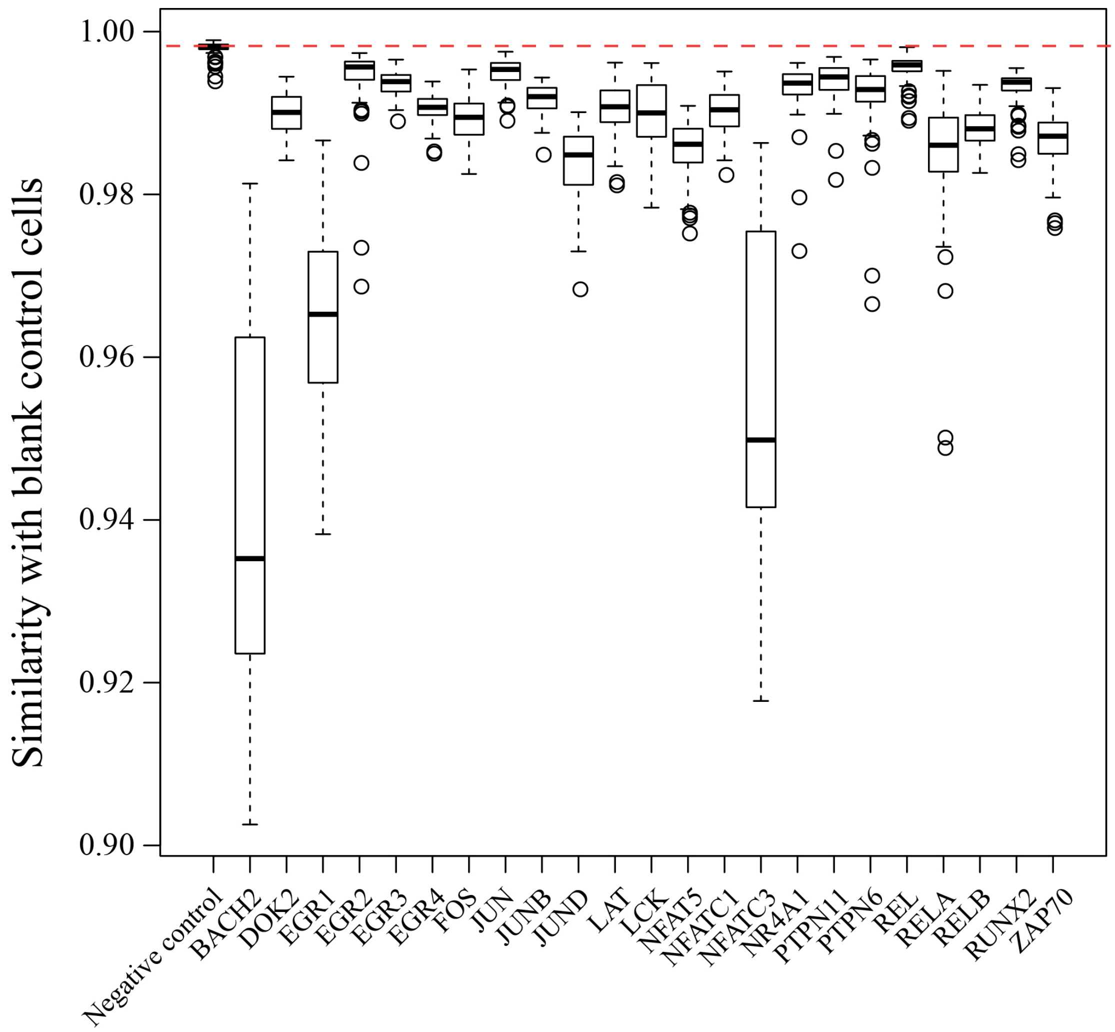

Supplementary Figure 15 Comparisons of blank control with negative control and other knockouts for stimulated Jurkat cell dataset.

Supplementary Table 1 Descriptions of all available single cell CRISPR screening datasets

Technology	GEO accession	Cell type	Species	Gene perturbation	No. of cells induced with sgRNAs	Experimental conditions
Perturb-Seq[4]	GSM2396856	BMDC	Mouse	24 TFs	21984	3 hr post-LPS
	GSM2396857	BMDC	Mouse	24 TFs	24589	0 hr post-LPS
	GSM2396858	K562	Human	10 TFs	33013	7 days post transduction
	GSM2396859	K562	Human	10 TFs	19268	13 days post transduction
	GSM2396860	K562	Human	10 TFs	51898	Higher MOI of perturbations
	GSM2396861	K562	Human	13 cell-cycle regulators	25971	-
Perturb-seq[3]	GSM2406675	K562	Human	7 TFs	5321	-
	GSM2406677	K562	Human	3 UPR related genes	13494	-
	GSM2406681	K562	Human	82 UPR related genes	50467	-
CRISP-seq[5]	GSE90486	Myeloid cells	Mouse	22 TFs	4977	4 hr post-LPS
CROP-seq[6]	GSM2439080 ~ GSM2439085	Jurkat cells	Human	6 regulators of TCR signaling and 23 TFs	3259	Stimulated by anti-CD3/CD28
	GSM2439086 ~ GSM2439090	Jurkat cells	Human	6 regulators of TCR signaling and 23 TFs	2646	-
Updated version of CROP-seq[7]	GSM2911346	MCF10A	Human	29 tumor suppressors	6283	Treated with doxorubicin
	GSM2911347	MCF10A	Human	29 tumor suppressors	6598	-

Supplementary Table 2 Comparison of gene relationships calculated with or without imputation, with or without filtering

Relationship	Prior knowledge	Data preprocessing strategies			
		None	Filtering only	Imputation only	Imputation+filtering
Runx1-Cebpb	Negative regulation	-0.03	-0.16	-0.67	-0.99
Irf4-Cebpb	Negative regulation	-0.1	-0.45	-0.63	-0.99
Nfkb1-Cebpb	Negative regulation	0.25	-0.32	-0.38	-0.99
Spi1-Cebpb	Negative regulation	0.3	-0.39	-0.67	-0.96
Junb-Cebpb	Positive regulation	0.69	0.07	0.37	0.93
Hif1a-Cebpb	Positive regulation	0.63	0.65	0.58	0.98
Stat3-Cebpb	Positive regulation	0.62	0.77	0.33	0.99
Rela-Cebpb	Positive regulation	0.77	0.89	0.54	0.99

Supplementary Table 3 Comparison of overall perturbation effect ranking identified by MUSIC or simply based on the number of differentially expressed genes. (DEG, differentially expressed genes calculated by Kolmogorov-Smirnov test. Perturb score, the TPDS defined in MUSIC as discussed in Methods)

Judging Criteria	Perturbation		
	PERK-IRE1	PERK-ATF6	PERK
Perturb score	109	103	93
No. Of DEG(p<0.05)	8414	8165	8386
No. Of DEG(p<0.01)	7899	7614	7816

Supplementary Table 4 Comparison of gene-by-gene correlations identified by MUSIC or by clustering RNA-seq profiles

Relationship	Prior knowledge	Strategies	
		Clustring RNA-seq profiles	MUSIC
Runx1-Cebpb	Negative regulation	0.57	-0.99
Irf4-Cebpb	Negative regulation	0.55	-0.99
Nfkb1-Cebpb	Negative regulation	0.55	-0.99
Spi1-Cebpb	Negative regulation	0.49	-0.96
Junb-Cebpb	Positive regulation	0.97	0.93
Hif1a-Cebpb	Positive regulation	0.92	0.98
Stat3-Cebpb	Positive regulation	0.97	0.99
Rela-Cebpb	Positive regulation	0.9	0.99
