# Supplementary material for: Model-based understanding of single-cell CRISPR screening
Source: Nat Commun. 2019 May 20;10:2233. doi: 10.1038/s41467-019-10216-x (PMC6527552; doi:10.1038/s41467-019-10216-x)
Supplement: Supplementary file 3 — Description of Additional Supplementary Files [file 41467_2019_10216_MOESM3_ESM.pdf]

Description of Additional Supplementary Files

Supplementary Data 1
Perturbation effect prioritizing result for BMDC with 3 hr post-LPS (GSM2396856).
Supplementary Data 2
Perturbation effect prioritizing result for unstimulated BMDC (GSM2396857).
Supplementary Data 3
Perturbation effect prioritizing result for K562 with 7 days post transduction (GSM2396858).
Supplementary Data 4
Perturbation effect prioritizing result for K562 with 13 days post transduction (GSM2396859).
Supplementary Data 5
Perturbation effect prioritizing result for K562 with high MOI (GSM2396860).
Supplementary Data 6
Perturbation effect prioritizing result for K562 with perturbation of cell cycle regulators (GSM2396861).
Supplementary Data 7
Perturbation effect prioritizing result for K562 cells (GSM2406675).
Supplementary Data 8
Perturbation effect prioritizing result for K562 with three UPR genes (GSM2406677).
Supplementary Data 9
Perturbation effect prioritizing result for K562 with the perturbation of 83 UPR genes (GSM2406681).
Supplementary Data 10
Perturbation effect prioritizing result for myeloid cells (GSE90486).
Supplementary Data 11
Perturbation effect prioritizing result for anti CD3/CD28 stimulated Jurkat cells (GSM2439080 ~ GSM2439085).
Supplementary Data 12
Perturbation effect prioritizing result for unstimulated Jurkat cells (GSM2439086 ~ GSM2439090).
Supplementary Data 13
Perturbation effect prioritizing result for doxorubicin treated MCF10A (GSM2911346).
Supplementary Data 14
Perturbation effect prioritizing result for untreated MCF10A cells (GSM2911347).
Supplementary Data 15
The proportion of filtered cells by filtering low efficiency sgRNA for all data sets.
Supplementary Data 16
Zero rate of all knockouts/knockdowns in all datasets.
Supplementary Data 17
Comparisons of overall perturbation effect ranking with or without imputation/filtering for all the available datasets.
